# The Meaning of Pain in the Clinical Management of Patients With Disorders of Consciousness: Is It a Neglected Issue?

**DOI:** 10.1002/ejp.70332

**Published:** 2026-07-06

**Authors:** Marianna Contrada, Giovanni Morone, Rocco Salvatore Calabrò, Federica Scarfone, Maria Elena Pugliese, Morena De Francesco, Chiara Milasi, Domenico Bosco, Maria Daniela Cortese, Lucia Francesca Lucca, Antonio Cerasa, Irene Ciancarelli, Francesco Riganello

**Affiliations:** ^1^ S. Anna Institute Crotone Italy; ^2^ Department of Life, Health and Environmental Sciences University of L'Aquila L'Aquila Italy; ^3^ Santa Lucia Foundation Scientific Institute for Research, Hospitalization and Health Care (IRCCS) Rome Italy; ^4^ Neurorehabilitation Unit IRCCS Neurolesi Center ‘Bonino‐Pulejo’ Messina Italy; ^5^ Institute of Bioimaging and Complex Biological Systems (IBSBC), National Research Council of Italy (CNR) Catanzaro Italy; ^6^ Neurology Department Pugliese Ciaccio Hospital Catanzaro Italy

## Abstract

**Background:**

Pain is a critical yet frequently underestimated component in the care of patients with acquired brain injury (ABI) and disorders of consciousness (DoC). Because these individuals often lack the ability to communicate verbally or purposefully, clinicians face substantial challenges in recognizing and managing pain, despite its profound influence on autonomic stability, behavioural responsiveness, rehabilitation engagement and overall prognosis.

**Content:**

This position paper synthesizes current knowledge on the multidimensional nature of pain and reviews the main behavioural, autonomic and neurophysiological tools available to assess nociception and pain‐related processing in non‐communicative patients. By integrating evidence from standard clinical scales with advanced physiological markers and neuroimaging findings, we highlight how preserved activity within the so‐called pain matrix challenges traditional assumptions about pain absence in DoC populations.

**Clinical Implications:**

The paper argues for a shift toward multimodal, patient‐centred approaches that combine behavioural observation with objective physiological indicators to improve diagnostic accuracy and ensure ethically responsible care. Practical guidance is provided on selecting appropriate assessment tools, interpreting behavioural and physiological signs of nociception, and implementing more effective pain management strategies.

**Conclusions:**

Accurate pain assessment is essential not only to promote recovery and facilitate rehabilitation but also to uphold the central ethical principle of safeguarding the dignity of patients with disorders of consciousness.

**Significance Statement:**

Understanding pain in Disorders of Consciousness (DoC) is a major clinical and ethical challenge, as residual nociceptive processing may be underestimated. This study advances a multidimensional framework integrating behavioural and neurophysiological evidence, moving beyond simple detection toward interpretation of pain meaning. It has direct implications for improving pain assessment, guiding personalized management and supporting more accurate and ethically grounded care in non‐communicative patients.

## 1 Introduction

Pain is a pervasive yet often underrecognized element of the clinical course after acquired brain injury (ABI), particularly in individuals with disorders of consciousness (DoC) (Chatelle, Thibaut, Whyte, et al. 2014; Schnakers et al. 2012). Rather than a simple sensory event, pain is a multidimensional experience involving sensory discriminative, affective and cognitive components that can influence rehabilitation participation and overall outcomes (Riganello et al. 2021). Despite its relevance, pain in DoC is still insufficiently assessed and frequently undertreated in routine clinical settings (Calabrò et al. 2021; Pistoia et al. 2016).

Disorders of consciousness (DoC) encompass a spectrum of conditions characterized by severely impaired arousal and awareness, ranging from coma to unresponsive wakefulness syndrome (UWS) and minimally conscious state (MCS). Over the past two decades, the conceptualization of DoC has shifted from a strictly behavioural taxonomy toward a multidimensional and graded framework of consciousness, reflecting increasing recognition that behavioural responsiveness is an imperfect proxy for underlying awareness (Kondziella et al. 2020; Giacino et al. 2018).

A key development in this field has been the identification of covert consciousness, including cognitive motor dissociation (CMD), in which patients demonstrate evidence of preserved volitional brain activity despite the absence of overt behavioural responses. While early neuroimaging studies provided proof of principle for this phenomenon (Owen et al. 2006; Monti et al. 2010), more recent large‐scale investigations have demonstrated that CMD may be present in a substantial proportion of patients lacking bedside command‐following, with some cohorts reporting prevalence rates approaching one quarter of clinically unresponsive individuals. These findings have major implications for diagnosis, prognosis and clinical decision‐making, as well as for the interpretation of residual cognitive and affective processing in DoC (Claassen et al. 2024; Kondziella et al. 2020). Crucially, this body of evidence reinforces the view that behavioural unresponsiveness cannot be equated with the absence of conscious experience, thereby highlighting the limitations of purely behaviour‐based diagnostics and supporting the adoption of multimodal assessment strategies.

In parallel, the clinical classification of DoC has undergone important refinement. The term UWS has progressively replaced ‘vegetative state’ to emphasize the presence of wakefulness without behavioural evidence of awareness (Laureys et al. 2010). Similarly, the MCS has been subdivided into MCS—and MCS+, reflecting different levels of behavioural complexity and, likely, different degrees of preserved conscious processing. MCS—is characterized by minimal but reproducible signs of awareness such as visual pursuit or localization to noxious stimulation, whereas MCS+ includes higher‐order behaviours such as command‐following, intelligible verbalization or functional communication (Bruno et al. 2011; Giacino et al. 2014; Golden et al. 2024). This distinction is clinically relevant, as MCS+ patients are more likely to demonstrate overt evidence of conscious awareness and, consequently, may have a higher probability of conscious pain experience, although pain perception cannot be excluded in MCS.

Contemporary international guidelines further support a multimodal diagnostic framework that moves beyond behavioural assessment alone. European and North American recommendations emphasize repeated and standardized clinical evaluations, systematic control of confounding factors such as sedation and sleep–wake cycle disturbances, and the integration of neurophysiological and neuroimaging techniques to improve diagnostic sensitivity and reduce misdiagnosis rates (Kondziella et al. 2020; Giacino et al. 2018). These advances underscore that DoC diagnosis should be understood as an iterative and multimodal process rather than a single behavioural classification.

These developments have major implications for the study of pain in DoC. While UWS has traditionally been associated with absent pain perception and MCS with partial awareness, emerging evidence shows that some non‐responsive patients exhibit cortical responses to nociceptive stimuli, suggesting the possibility of nociceptive processing, but not necessarily a high probability of conscious pain experience. Taken together, these findings support a shift toward a consciousness‐informed framework for pain assessment, in which nociceptive responses are interpreted within the broader context of the patient's capacity for sustained and integrated conscious processing. In this perspective, pain cannot be inferred solely from behavioural output or physiological reactivity, but must be probabilistically assessed through the integration of multimodal markers of both nociception and consciousness capacity.

Pain also has substantial implications for rehabilitation trajectories. Uncontrolled pain can worsen spasticity, trigger maladaptive physiological responses such as paroxysmal sympathetic hyperactivity and diminish the patient's ability to engage in therapeutic activities, ultimately delaying recovery of consciousness (Chatelle et al. 2012; Chatelle et al. 2018; Baguley et al. 2014). In contrast, effective pain management has been associated with improved participation and more favourable functional outcomes (Calabrò et al. 2021; Papic et al. 2025). Addressing pain should therefore be a priority throughout the continuum of ABI recovery, not solely during the DoC phase.

Recognizing pain as more than a reflex is crucial in patients unable to communicate. Its presence may indicate residual or covert awareness, carrying important prognostic and ethical implications. Pain should thus be conceptualized as a dynamic, multidimensional phenomenon requiring integrated, interdisciplinary assessment and management strategies (Boly et al. 2008).

This paper aims to critically examine current clinical approaches to pain assessment in DoC, compare the most used scales, outline their limitations and explore how neurophysiological advances may enhance diagnostic and therapeutic decision‐making.

## 2 Conceptual Framework of Pain in DoC

### 2.1 Defining Pain: Biological, Physiopathological and Psychological Dimensions

Pain is the physiologic manifestation to the detection of noxious stimuli by nociceptors, called nociception. Nociception is ‘an actual or potentially tissue damaging event transduced and encoded by nociceptors’ while pain constitutes a first‐person experience (Loeser and Treede 2008). Pain itself may not always be linked to a stimulus and does not necessarily correlate with the degree of injury severity (Lee and Neumeister 2020). Indeed, pain cannot be reduced to the perception of a harmful stimulus, as it involves biological and psychological processes. In addition to its multidimensional nature, contemporary classifications of pain distinguish between nociceptive, neuropathic and nociplastic mechanisms. Nociceptive pain results from the activation of peripheral nociceptors by actual or threatened tissue damage, whereas neuropathic pain is caused by a lesion or disease of the somatosensory nervous system. More recently, the International Association for the Study of Pain has introduced the concept of nociplastic pain, defined as pain arising from altered nociception despite no clear evidence of tissue damage or somatosensory system lesion (Raja et al. 2020). This category is particularly relevant in ABI, where central nervous system dysfunction may lead to altered sensory processing, central sensitization and maladaptive network activity. In such cases, pain may emerge from dysregulated interactions within large‐scale brain systems, including salience, affective and cognitive networks, rather than from direct peripheral or structural neural damage. In patients with DoC, distinguishing among these mechanisms is especially challenging, as behavioural output is severely impaired and subjective report is absent. Consequently, the underlying nature of pain—whether nociceptive, neuropathic or nociplastic—must be inferred indirectly through multimodal assessment, further reinforcing the need for an integrated framework that combines physiological, neurophysiological and neuroimaging markers.

From a biological perspective, pain depends on complex mechanisms of nerve transduction and modulation, and neuroplasticity can contribute to maintaining the sensation of pain even in the absence of obvious tissue damage (Dehghan et al. 2024). At the same time, emotional and cognitive factors, as well as the personal meaning attributed to suffering, profoundly modulate the subjective experience, particularly in patients with acquired brain injuries (ABI), where pain can exacerbate physical and cognitive disabilities and negatively affect functional recovery and quality of life (Juárez‐Belaúnde et al. 2025). In this framework, the transition from peripheral detection to central integration is mediated by a distributed cortico–subcortical network known as the *pain matrix* (Iannetti and Mouraux 2010; Salomons et al. 2016). However, the transition from peripheral nociception to pain should not be understood as the activation of a pain‐specific neural module. Historically, the term ‘pain matrix’ has been used to describe a distributed set of cortical and subcortical regions commonly recruited by nociceptive stimulation, including the thalamus, primary and secondary somatosensory cortices, insula, anterior/mid‐cingulate cortex, prefrontal regions and brainstem modulatory structures (Legrain et al. 2011; Salomons et al. 2016). However, this network should not be interpreted as a stable or pain‐specific biomarker of conscious pain perception. Evidence from neuroimaging and electrophysiological studies indicates that many of these regions are also activated by salient non‐painful stimuli, attentional orienting, interoceptive changes, motor preparation, threat detection and cognitive control demands (Menon and Uddin 2010; Legrain et al. 2011; Shackman et al. 2011). Accordingly, the so‐called pain matrix is better conceptualized as a set of partially overlapping sensory‐discriminative, affective‐motivational, cognitive‐evaluative, salience‐related and autonomic/modulatory processes through which nociceptive input may contribute to conscious pain experience when sufficient integrative conditions are preserved.

Pain processing is therefore supported by a distributed and dynamically interacting architecture. Ascending nociceptive information reaches lateral thalamic nuclei and somatosensory cortices, supporting sensory‐discriminative processing (Lenz et al. 2024; Motzkin et al. 2024), while medial thalamic, parabrachial, insular, cingulate, amygdala, hypothalamic and striatal circuits contribute to salience detection, affective‐motivational value, defensive relevance and autonomic arousal (Legrain et al. 2011). Cognitive‐evaluative networks, including prefrontal orbitofrontal, lateral parietal, hippocampal and default‐mode regions, contextualize nociceptive input in relation to expectations, memory, attention, perceived threat, controllability and self‐related meaning (Wiech 2016; Tajerian et al. 2018). These cortical and limbic systems interact bidirectionally with descending modulatory pathways centered on the periaqueductal grey, rostral ventromedial medulla and locus coeruleus, thereby shaping spinal nociceptive transmission through inhibitory or facilitatory control (Ossipov et al. 2014). The cerebellum should be considered an additional predictive sensorimotor‐autonomic node, contributing to timing, adaptation, defensive motor preparation, autonomic adjustment and pain‐related modulation rather than merely reflecting motor output (Li et al. 2024).

**FIGURE 1:**
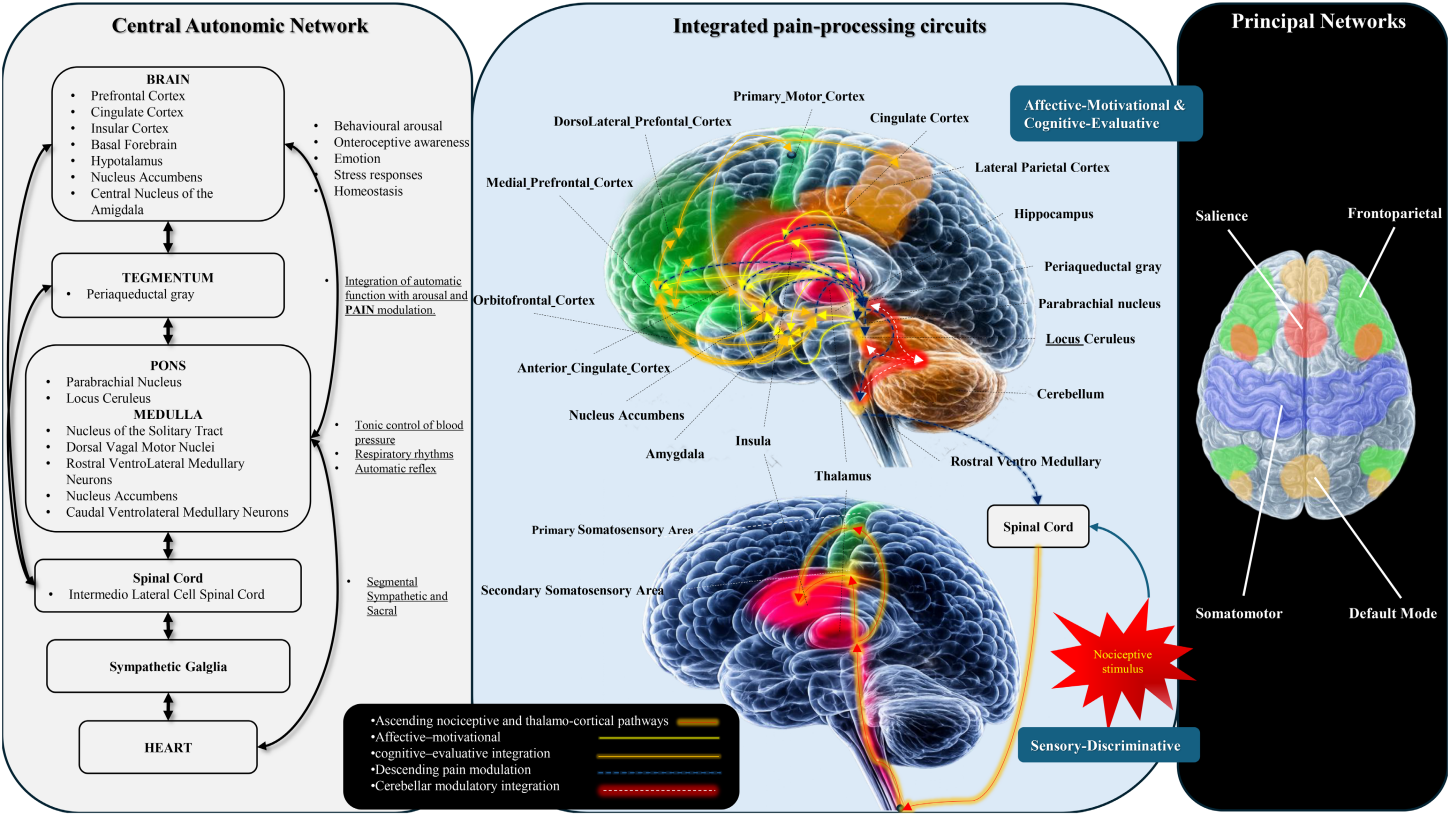
Nociceptive, pain‐related and autonomic circuits embedded within the Central Autonomic Network. The figure illustrates pain‐related processing as a distributed architecture. The left panel summarizes the Central Autonomic Network, including cortical, subcortical, brainstem, spinal, sympathetic and cardiac levels. The central panel separates the sensory‐discriminative nociceptive processing from affective‐motivational, cognitive‐evaluative, descending modulatory and cerebellar modulatory integration. Yellow/red arrows indicate ascending nociceptive and thalamo‐cortical pathways, including spinal dorsal horn processing, anterolateral/spinothalamic transmission, thalamic relay and projections to S1/S2 and posterior insula. Yellow and orange arrows indicate affective‐motivational and cognitive–evaluative integration among prefrontal, cingulate, insular, amygdala, hippocampal, striatal and lateral parietal regions. Dashed blue arrows indicate cortico‐limbic‐brainstem descending pain modulation through PAG, RVM/LC and spinal dorsal horn. Dashed white/red‐halo arrows indicate cerebellar modulatory integration, reflecting the cerebellum's proposed role in prediction, timing, sensorimotor adaptation, autonomic adjustment and interaction with brainstem modulatory systems. The right panel summarizes large‐scale cortical networks involved in salience, somatomotor, frontoparietal and default‐mode processing.

This distinction is particularly relevant in patients with DoC, in whom preserved nociceptive responses may indicate residual afferent processing, salience detection, autonomic reactivity or partial cortical integration, but do not necessarily demonstrate conscious pain (Laureys et al. 2002; de Tommaso et al. 2015). Pain‐related findings in DoC should therefore be interpreted probabilistically and multimodally, rather than as direct evidence of subjective experience. This requirement becomes even more critical considering recent evidence indicating that large‐scale brain dynamics, thalamocortical connectivity and measures of neural complexity may provide key insights into the capacity for conscious experience in behaviorally unresponsive patients. Accordingly, nociceptive processing should be interpreted in relation to the integrity of such large‐scale integrative mechanisms. Within this revised framework, the Central Autonomic Network (CAN) provides a useful integrative architecture because several pain‐related nodes overlap with autonomic and interoceptive regulatory circuitry, including the insula, anterior/mid‐cingulate cortex, prefrontal cortex, amygdala, hypothalamus, PAG, brainstem nuclei, spinal autonomic pathways and peripheral cardiovascular targets (Riganello et al. 2019; Cortese et al. 2020). Figure 1 summarizes this revised view by distinguishing sensory‐discriminative nociceptive pathways from affective‐motivational, cognitive‐evaluative, descending modulatory, cerebellar and autonomic components embedded within the broader CAN. Importantly, this perspective implies a hierarchical organization of pain processing in which nociceptive input alone is not sufficient to generate conscious pain. Rather, conscious pain experience emerges only when nociceptive signals are integrated across large‐scale brain networks supporting awareness, salience attribution and self‐related processing. In conditions where such integrative capacity is severely disrupted, as in disorders of consciousness, nociceptive processing may persist in the absence of subjective pain. Accordingly, pain in DoC should be understood as a probabilistic phenomenon, whose likelihood depends on the degree of preserved large‐scale brain integration rather than on the mere presence of nociceptive responses.

Beyond nociceptive input and its central processing, pain is shaped by psychological mechanisms that determine how bodily signals are selected, interpreted and responded to. Contemporary models converge in describing at least three tightly interacting psychological components. (i) Cognitive components include attentional allocation to bodily threat, appraisal and meaning attribution (‘What is happening to me?’), expectations and predictions about pain, perceived controllability and learning processes that update threat value and safety signals. These mechanisms influence both pain intensity and unpleasantness and contribute to sensitization or, conversely, to adaptive down‐regulation. (ii) Affective–motivational components capture the unpleasantness of pain, its salience and threat value and associated emotions such as fear, anxiety, anger or helplessness that can promote avoidance and hypervigilance. (iii) Behavioural and communicative components include protective/avoidance behaviours, facial and vocal expression, agitation/restlessness and interpersonal communication of suffering, which are critical in clinical contexts where self‐report is limited. Importantly, these components may be partially dissociable: observable behaviour can be reduced by motor impairment or sedation despite ongoing nociceptive drive, and conversely agitation can occur without pain, complicating inference in DoC. These domains are consistent with the biopsychosocial formulation of pain communication proposed by Hadjistavropoulos et al. (2011), which similarly emphasizes cognitive, affective‐emotional and behavioural‐communicative processes.

## 3 The Management of Pain in Clinical Practice of DoC

From a clinical perspective, multiple sources of pain should be systematically considered. In the acute phase following ABI, pain may result from fractures, soft tissue injuries, surgical wounds, invasive devices (e.g., nasogastric tubes, urinary catheters) and early neuropathic mechanisms (Barr et al. 2013; Schnakers and Zasler 2015). During the subsequent rehabilitation phase, common contributors include spasticity, contractures, heterotopic ossification, joint and musculoskeletal disorders, central (thalamic) pain, pressure ulcers and visceral complications such as constipation (Gironda et al. 2009; Thibaut et al. 2015).

In addition to these well‐established causes, contemporary pain frameworks suggest that nociplastic mechanisms may also play a relevant role in ABI populations (Juárez‐Belaúnde et al. 2025). Central sensitization altered thalamocortical processing and dysfunctional large‐scale network integration may generate persistent pain‐like states even in the absence of ongoing tissue damage or overt somatosensory lesions. Furthermore, although difficult to assess, factors such as distress related to anticipatory responses to care procedures, affective dysregulation or depressive symptomatology may contribute to pain‐related suffering and should be considered in clinical interpretation. Overall, a clinically oriented approach for the management of pain in DoC patients should consider (Wang et al., 2024):

### 3.1 Principles of Pain Management in DoC

Effective pain management in DoC requires a multidimensional and iterative approach that integrates clinical observation, physiological monitoring and individualized therapeutic strategies. Given the absence of verbal report, clinicians must rely on a combination of behavioural indicators, autonomic responses and contextual clinical information to guide treatment decisions. From a practical standpoint, pain management should follow four core principles:

- Etiological identification: whenever possible, clinicians should identify and directly treat underlying causes of pain (e.g., correcting joint malalignment, managing infections, adjusting medical devices).
- Multimodal assessment: behavioural scales (e.g., NCS‐R) should be combined with physiological and clinical indicators to improve sensitivity and reduce misinterpretation.
- Treatment Individualization: treatment strategies should be tailored to the patient's clinical condition, neurological status and suspected pain mechanism.
- Longitudinal monitoring: changes over time, particularly before and after interventions, are critical for interpreting pain‐related responses and treatment efficacy.

### 3.2 Pharmacological Strategies

Pharmacological therapy is not always necessary in this population, because a careful early identification of removable causes of distress always needs to be addressed (uncomfortable position, urinary catheter obstruction, in example). Overall, analgesic therapy should be guided by the suspected underlying mechanism:

- Nociceptive pain: non‐opioid analgesics (e.g., paracetamol, NSAIDs) and, when necessary, opioids may be used, paying attention to sedation effects that may interfere with behavioural assessment.
- Neuropathic pain: agents such as gabapentinoids, antidepressants or other neuromodulators should be considered based on clinical suspicion.
- Spasticity‐related pain: antispastic treatments (e.g., baclofen, botulinum toxin injections) may reduce both motor impairment and associated discomfort.
- Nociplastic‐like pain: centrally acting drugs targeting network‐level dysregulation may be considered, although evidence in DoC populations remains limited.

Importantly, pharmacological interventions should be introduced cautiously, with gradual titration and careful monitoring to avoid confounding effects on arousal, responsiveness and autonomic regulation.

### 3.3 Monitoring and Evaluation of Treatment Effects

One of the main challenges in DoC is determining whether an intervention has effectively reduced pain. Since subjective feedback is unavailable, treatment evaluation must rely on indirect indicators. Clinicians should systematically assess:

- changes in behavioural responses (e.g., reduced grimacing, agitation, defensive movements)
- modifications in autonomic signals (e.g., heart rate variability, skin conductance, pupillary response)
- variations in neuronal or electrophysiological markers, when available
- overall clinical stability and tolerance to care procedures

A particularly informative approach is repeated measurement before and after analgesic administration, allowing clinicians to infer the functional relevance of observed responses. This ‘therapeutic trial’ approach is especially useful in distinguishing nociceptive‐driven behaviours from nonspecific arousal or environmental reactivity.

### 3.4 Ethical Considerations

Pain management in DoC is not only a clinical issue, but also a central ethical responsibility. The inability to communicate does not imply the absence of subjective experience and the risk of both undertreatment and overtreatment must be carefully balanced. From an ethical perspective:

- clinicians should adopt a precautionary principle, assuming the possibility of pain when uncertainty persists.
- painful procedures should be minimized, standardized and clinically justified
- treatment decisions should consider potential effects on consciousness assessment, avoiding excessive sedation that may obscure residual awareness
- interdisciplinary collaboration, including caregivers and family members, may provide additional insight into patient‐specific responses.

Ultimately, pain management in DoC requires navigating uncertainty while preserving patient dignity, minimizing suffering and avoiding both neglect and overinterpretation of indirect signals (Fins and Bernat 2018).

## 4 Assessment of Pain: Clinical Measures

Table 1 provides a structured overview of the main tools used to assess nociception and pain‐related processing across DoC phenotypes. Importantly, a critical distinction must be maintained: these instruments do not directly measure subjective pain but rather assess behavioural and physiological responses to nociceptive stimulation. In the absence of self‐report, these responses represent indirect markers that may be compatible with pain experience but cannot be considered definitive evidence of conscious pain. Consequently, pain assessment in DoC should be understood as an inferential and probabilistic process, in which clinical interpretation depends on the integration of behavioural, physiological and contextual information.

**TABLE 1:** The main clinical scales employed to subjectively measure pain in DoC patients.

Scale	Aim's study	Study's design and population	Assessment	Results
PAINAD (Fry et al., 2018)	Assessing pain in individuals with advanced dementia	No verbal patients with advanced dementia, also adapted for GCA	Behavioural pain is assessed using five indicators breathing, vocalization, facial expression, body language and consolability. Each category can be given a score from 0 (not present) to 2 (fully present)	The PAINAD was created by reviewing the literature and incorporating existing frameworks for measuring pain, such as the FLACC scale. It is based on direct observation rather than patient self‐reporting and is defined by letteratura as a valid (accurate) and reliable (consistent) indicator of pain in people with cognitive impairments
BPS (Payen et al. 2001)	Assess non‐verbal patients' pain, sedated or unable of verbally expressing their pain	170 patients' pain, in intensive care units (ICUs) and who are sedated or unable of verbally expressing their pain	A behavioural scale used in non‐responsive patients undergoing mechanical ventilation that assesses facial expression, upper limb movements and compliance with mechanical ventilation. The BPS score ranges from 3 to 12, with each category being evaluated on a scale of 1–4	The patients reported no pain when they were at rest, mild discomfort when they were shifting positions and receiving respiratory physical therapy, mild to moderate discomfort when they were washing their mouths, and significant discomfort when they were sucking secretions. Pairwise comparisons of pain scores in various contexts using the Wilcoxon test revealed a significant difference in both scales. For all operations, there were robust and positive associations between the BPS and CPOT pain scores of ICU‐admitted patients
CPOT (Gélinas et al. 2006)	Assess CPOT in patients who have undergone cardiac surgery, including those who are unconscious/intubated	105 patients undergoing heart surgery were admitted to the intensive care unit. 33 out of the 105 patients were assessed while unconscious and intubated after surgery, and 99 were assessed while aware and intubated; all 105 patients were assessed following extubation	Patients were assessed using the Critical‐Care Pain Observation Tool at rest, during a nociceptive procedure (positioning) and 20 min post‐procedure, for a total of nine assessments, for each of the three testing periods. Pain was self‐reported by each patient both during and after intubation while they were conscious	Validity and sensitivity in identifying pain during nociceptive procedures
NCS (Schnakers et al. 2010)	Validating a scale for nociception in patients with consciousness disorders (VS, MCS)	48 post‐coma patients (28 VS, 20 MCS)	NCS was created especially to evaluate how patients with pDoCs respond to pain stimulation in their motor, verbal, facial and visual behaviours and to separate reflex from higher‐level actions in response to painful stimuli and other external stimuli	Significant differences between MCS and VS, good inter‐observer agreement and good concurrent validity
NCS‐R (Chatelle et al. 2012)	To determine the sensitivity of the Nociception Coma Scale (NCS) in detecting behavioural changes in response to noxious versus non‐noxious stimulation in patients with vegetative state and minimally conscious state	Observational study comparing behavioural responses across different stimulation conditions; in 64 patients with disorders of consciousness	NCS administered under three conditions: baseline (spontaneous behaviour), non‐noxious tactile stimulation (shoulder taps) and noxious stimulation (pressure on the nail bed)	NCS total scores and motor, verbal and facial subscores were significantly higher during noxious stimulation compared to non‐noxious stimulation. No significant difference was observed for the visual subscale. A cut‐off score of 4 differentiated noxious from non‐noxious stimulation; exclusion of the visual subscale increased sensitivity, with high specificity and accuracy
NCS‐R (Formisano et al. 2020, 2024)	To compare Nociception Coma Scale–Revised scores obtained with standard painful stimulation (NCS‐R‐SS) versus personalized painful stimulation (NCS‐R‐PS) in persons with disorders of consciousness and to assess convergent validity with the Coma Recovery Scale–Revised (CRS‐R)	Prospective, international, multicenter observational study; 61 adults with prolonged disorders of consciousness	Standard stimulus (fingernail pressure) and personalized painful stimulus identified by caregivers and clinicians; level of consciousness assessed using CRS‐R	NCS‐R‐PS scores were significantly higher than NCS‐R‐SS scores, showing greater sensitivity. Significant correlations were found between NCS‐R‐PS and NCS‐R‐SS (concurrent validity) and between NCS‐R‐PS and CRS‐R at all time points. Reliability was acceptable to good, with slightly better psychometric performance for personalized stimuli.
BINAM (White et al., 2020)	Measuring nociception in non‐communicative patients with severe TBI	157 patients with severe brain injury	10‐item observational scale of behavioural and physiological indicators (facial expressions, movements, tone, cardiovascular parameters)	The scale shows good sensitivity to pain/nociception, with scores increasing with nociceptive activities (e.g., physiotherapy); good response to analgesia, with scores decreasing with paracetamol; it can be used regardless of the level of consciousness or agitation; and it has good personal reliability, robust to missing data.

Abbreviations: BINAM, Brain Injury Nociception Assessment Measure; BPS, Behavioural Pain Scale; CPOT, Critical‐Care Pain Observation Tool; CRS‐R, Coma Recovery Scale–Revised; NCS, Nociception Coma Scale; NCS‐R, Nociception Coma Scale‐Revised; PAINAD, Pain Assessment in Advanced Dementia Scale.

From a conceptual perspective, available tools can be broadly categorized according to the dimension of nociceptive processing they emphasize:

- affective–behavioural indicators (e.g., PAINAD), which capture distress‐related expressions;
- motor–defensive reactivity (e.g., BPS, CPOT), reflecting reflexive responses to nociceptive input;
- DoC‐specific behavioural nociception (e.g., NCS, NCS‐R), designed to differentiate reflexive and potentially higher‐order responses; and
- physiological/autonomic indices (e.g., BINAM), aiming to quantify nociceptive drive independently of motor output or awareness.

This classification highlights that different instruments capture distinct components of nociceptive processing and therefore provide complementary, rather than interchangeable, information.

The Pain Assessment in Advanced Dementia (PAINAD) scale (Warden et al. 2003), the Nociception Coma Scale (NCS) (Schnakers et al. 2010; Sattin et al. 2018), and its updated version, the Nociception Coma Scale–Revised (NCS‐R) (Chatelle et al. 2012; Formisano et al. 2020, 2024), are among the most commonly used instruments for evaluating patients with sABI and those in a Minimally Conscious State (MCS). The NCS was specifically designed to assess motor, verbal, facial and visual responses to pain stimuli in patients with prolonged Disorders of Consciousness (pDoCs), aiming to distinguish between reflexive and higher‐order behavioural reactions to external stimuli, including noxious inputs. A revised version, the NCS‐R, was later introduced, excluding the visual subscale, as this component was found to lack sensitivity in detecting responses to painful stimuli in this patient population. The NCS‐R has also been further investigated to evaluate behavioural reactivity to nociceptive stimulation and estimate the likelihood of preserved pain‐related processing (NCS‐R‐SS) and personalized stimuli (NCS‐R‐PS). Several studies have confirmed the superiority and validity of the personalized pain stimulus approach over the standardized methodology in assessing pain in individuals with Disorders of Consciousness (DoC). PAINAD is a clinically relevant and user‐friendly observational tool designed to assess pain in adults with advanced dementia who are nonverbal or have severely limited self‐reporting abilities. Rather than requiring patients to provide a pain score, it relies on caregivers or physicians to evaluate specific behavioural indicators. Less commonly used but equally useful scales are the Critical‐Care Pain Observation Tool (CPOT) and the Behavioural Pain Scale (BPS). Gélinas et al. (2006) validated the Critical‐Care Pain Observation Tool (CPOT) as a key instrument for evaluating pain in intubated and non‐communicative patients. The CPOT is based on behavioural indicators and has demonstrated good reliability and clinical utility. However, its implementation requires specific training to ensure accurate and consistent scoring by healthcare professionals. Similarly, Payen et al. (2001) examined the use of the Behavioural Pain Scale (BPS) in unresponsive ICU patients. The BPS has been shown to be effective in supporting analgesic decision‐making in critically ill patients. Nevertheless, its accuracy may be compromised in the presence of sedative agents or neuromuscular blockade, and like the CPOT, it necessitates adequate staff training for optimal use.

Among the tools recently proposed for assessing nociceptive response in patients with severe non‐communicative brain injury is the Brain Injury Nociception Assessment Measure (BINAM), developed by Whyte et al. (2020) through an item selection process based on Item Response Theory. The scale includes 10 behavioural and physiological indicators designed to measure nociception independently of the level of consciousness or agitation. BINAM has shown good sensitivity to expected clinical changes, with scores increasing in the presence of potentially painful stimuli and decreasing after the administration of analgesics, confirming its reliability and relevance for monitoring these patients. These instruments are not designed for patients with consciousness disorders. However, they may be used in clinical or experimental settings if used with caution.

It is important to note that the most used clinical scales for assessing responsiveness in patients with DoC also include nociceptive (i.e., pain‐related) stimuli as part of the evaluation. However, these instruments do not measure pain itself; rather, they assess the patient's behavioural responses to potentially painful stimuli in order to determine their level of consciousness. For instance, the Simplified Evaluation of Consciousness Disorders (SECONDs; Aubinet et al. 2021) incorporates nociceptive stimulation within its assessment protocol. Specifically, it includes a conditional item called ‘localization to pain,’ which is administered when the patient does not meet predetermined thresholds on other behavioural items. The Coma/Near‐Coma Scale (CNC; Sandberg 2011) specifically includes a nociceptive stimulus item, distinct from tactile stimulation. The stimulus is applied following a controlled protocol (e.g., nail bed pressure, pinch), and the quality of the response is observed. The Full Outline of UnResponsiveness (FOUR; Iyer et al. 2009) in the motor domain, uses painful stimuli to elicit a response when commands cannot be followed. Pain is not directly assessed but serves as a standardized nociceptive stimulus to determine motor responsiveness (e.g., localisation, decortication, decerebration or no response). The Sensory Modality Assessment and Rehabilitation Technique (SMART; Gill‐Thwaites and Munday 2004) does not assess subjective pain either but instead evaluates whether there is a response to a nociceptive stimulus, what type of response occurs (e.g., reflexive, localized, discriminative) and how consistent it is over time. The Coma Recovery Scale‐Revised (CRS‐R) (Giacino 2004), which is considered the gold standard for monitoring disorders of consciousness, includes painful stimuli as part of its assessment. It does not assess subjective pain perception but instead measures behavioural responses to nociceptive stimuli to evaluate the level of consciousness. The pain stimulus is included in the motor subscale and is also used as part of the arousal evaluation. The Wessex Head Injury Matrix (WHIM; Shiel et al. 2000) is not a stimulus‐based structured scale like the CRS‐R or SMART. However, in clinical application, tactile or nociceptive stimuli may be used, especially to observe behaviours in the lower levels of the scale. Unlike the other scales, WHIM does not specify which stimulus to use for each item—it is up to the clinician to choose the most appropriate stimulus and record the behaviour observed. Similarly, the Western Neuro Sensory Stimulation Profile (WNSSP; Ansell and Keenan 1989) observes if and how the patient responds to a painful stimulus, and assigns a score based on that response (see Table 2).

**TABLE 2:** Sub‐clinical scales most commonly used for assessing pain.

Scale	Uses pain stimulus?	Type of stimulus	Assesses subjective pain perception?	Type of response assessed	Role of pain in the scale
SECONDs (Aubinet et al. 2021)	Yes (conditional)	Nail bed pressure	No	Pain localization (observational)	Used only if other items are negative; helps detect MCS
CNC (Sandberg 2011)	Yes	Standardized noxious stimuli	No	0 (no response) to 4 (extreme response)	Assesses depth of coma or near‐coma
FOUR (Iyer et al. 2009)	Yes	Not a single method	No	Motor scale from 0 (no response to pain) to 4 (voluntary movements/obeys commands)	Test motor function and brainstem responsiveness, determine the degree of neurological impairment.
SMART (Gill‐Thwaites and Munday 2004)	Yes	Controlled noxious stimuli (e.g., pinch)	No	5‐level scale: from no response to discriminative response	Multisensory profile for assessment and rehab
CRS‐R (Giacino 2004)	Yes	Nail bed pressure, pinch	No	Reflex, withdrawal, localization, posture	Determines level of consciousness (e.g., VS vs. MCS)
WHIM (Shiel et al. 2000)	Yes*	Painful stimulus may be used to elicit behaviours	No	Observational behaviours (withdrawal, localization)	Tracks recovery in a hierarchical behavioural order
WNSSP (Ansell and Keenan 1989)	Yes	Pressure on the nail bed, trapezius stimulation or deep pressure, joint stimulation	No	Stimulus localisation, withdrawal/avoidance, appropriate movements vs. reflexes, level of alertness associated with the response, consistency of responses over time (motor and sensory patterns)	Sensory integrity and arousal level

Abbreviations: CNC, Coma/Near Coma Scale; CRS‐R, Coma Recovery Scale‐Revised; FOUR, Full Outline of UnResponsiveness; SECONDs, Simplified Evaluation of Consciousness Disorders; SMART, Sensory Modality Assessment and Rehabilitation Technique; WHIM, Wessex Head Injury Matrix; WNSSP, Western Neuro Sensory Stimulation Profile.

*Test not standardized.

Across heterogeneous clinical populations, from advanced dementia to DoC, the assessment of nociception relies on operationalizing observable behaviours and physiological correlates that act as indirect markers of pain processing. Although each scale arises from a distinct theoretical lineage, they can be conceptualized as occupying different positions along a continuum whose poles are affective–behavioural distress, defensive motor reactivity and physiological nociceptive drive. PAINAD represents the most affectively weighted model, rooted in the assumption that late‐stage dementia patients preserve fragments of expressive affective behaviour (Warden et al. 2003). Its domains (vocalization, respiration, consolability, facial expression) emphasize autonomic–affective dysregulation rather than nociception in a strict neurophysiological sense. Here, nociception is inferred primarily through behavioural disorganization, making the scale sensitive but poorly specific for DoC, where affective expressivity is largely uncoupled from cortical integration (Table 3).

**TABLE 3:** Comparative framework of pain/nociception scales in non‐communicative patients.

Scale	Implicit theoretical/conceptual model	Observed domains	Prevalent clinical meaning	Inferred functional processes	Strengths	Interpretative limitations	Optimal context of use
PAINAD (Warden et al. 2003)	Affective–behavioural model; nociception as affective–vegetative disorganization in advanced cognitive impairment	Vocalizations, facial expression, respiration, body language, consolability	Residual affective expressivity as an indirect indicator of nociception in severe dementia	Autonomic dysregulation; emotional–visceral activation	Fast, intuitive, widely used in advanced dementia	Very low specificity in DoC; conflates nociception with non‐nociceptive distress	Advanced dementia; geriatric settings; not recommended as primary tool in DoC
BPS (Payen et al. 2001)	Defensive behavioural model; nociception as graded biomechanical defensive response	Facial grimacing; limb movements; ventilatory pattern	Progressive defensive response to nociceptive or procedural stimuli	Segmental and brainstem defensive reflexes; ventilatory adaptations	Standardized, simple; useful for procedural pain	Ambiguous in DoC; low sensitivity under deep sedation or neuromuscular blockade	ICU; invasive procedures in intubated or deeply sedated patients
CPOT (Gélinas et al. 2006)	Integrated behavioural–physiological model; nociception as perturbation of the procedural homeostasis	Facial expression; body movements; muscle tension; ventilator compliance	Nociception detected by integrating motor output and autonomic/ventilatory reactivity	Motor activation; modulation of respiratory drive and thoraco‐abdominal mechanics	Extensively validated; good discrimination of procedural pain; robust in ICU	Requires residual motor output; influenced by sedation depth and ventilator settings	ICU, especially intubated and mechanically ventilated patients
NCS (Schnakers et al. 2010)	Nociceptive–physiological model; nociception as ‘raw’ behavioural reactivity in DoC	Facial response; motor response; verbal/vocal response; visual response	Behavioural reactivity to nociceptive stimuli specifically in DoC	Spinal and bulbospinal reflexes plus possible thalamo‐limbic activation	First scale specifically designed for DoC; good specificity for nociception vs. background motor activity	Limited distinction between reflexive and cortically integrated responses; visual subscale later found less sensitive	DoC screening; serial monitoring of nociceptive reactivity in post‐acute and chronic DoC
NCS‐R (STANDARD STIMULUS, SS) (Chatelle et al. 2012)	Standardized nociceptive model; improved signal‐to‐noise ratio for bedside nociception in DoC	As NCS, but without the visual item: facial, motor and verbal/vocal response to a uniform nail‐bed pressure	Behavioural response to a controlled, replicable nociceptive stimulus, optimized for comparability across raters and sessions	Spinal/bulbospinal nociceptive reflexes with higher probability of thalamo‐limbic and anterior cingulate engagement	Better psychometric properties than original NCS; high reproducibility; recommended reference for DoC nociception	Underestimates nociception in patients with peripheral hypoesthesia, focal deficits or atypical pain generators; ceiling/floor effects in some profiles	Clinical and research use in DoC; serial monitoring; protocolized nociception assessment in guidelines and trials
NCS‐R (Personalized stimulus, PS) (Formisano et al. 2020, 2024)	Contextualized nociceptive model; ‘situated embodiment’ where nociception is elicited by patient‐specific clinical pain sources	Same behavioural items as NCS‐R, but in response to individualized stimuli derived from clinical observation (spasticity, heterotopic ossification, pressure ulcers, devices, etc.)	Enhanced detection of clinically relevant nociception; higher sensitivity to residual pain perception and covert reactivity compared with standard stimulus	Peripheral sensitization (Aδ/C fibres); spinal plasticity; increased likelihood of recruiting thalamo‐limbic and cingulate circuits by ecologically salient stimuli	Maximizes sensitivity to real‐life pain generators; better ecological validity; stronger correlations with CRS‐R and with clinically observed pain behaviours	Lower standardization; requires expertise and careful protocolization; more vulnerable to inter‐site heterogeneity and bias in choice of stimulus	Advanced DoC assessment; international multicenter protocols; situations where NCS‐R‐SS scores are low but clinical suspicion of pain remains high
BINAM/BIDAL (Whyte et al. 2020)	Unidimensional nociceptive model derived from item‐response theory; nociception as a latent bottom‐up physiological drive, designed to be independent of level of consciousness and agitation	Ocular arousal; cardiovascular parameters (systolic/diastolic BP, HR); pupil diameter; facial expression; posture; non‐goal‐directed movements; primitive vocalizations; skin colour	Quantification of *true* nociceptive load in non‐communicative patients, largely independent of consciousness state and psychomotor agitation; sensitive to activity and analgesia	Sympatho‐adrenergic neurovegetative activation; brainstem reflexes; modulation of postural tone; subcortical arousal variation; non‐cortical and possibly cortical nociception‐related motor output	Explicit independence from level of consciousness and agitation; strong psychometric robustness (Rasch/IRT); integrated physiological–behavioural construct; feasible even in highly agitated severe TBI	Cardiovascular data may be unavailable/unreliable in very mobile or hemodynamically unstable patients; reduced utility in severe oculomotor deficits; requires repeated/continuous observation	Acute and subacute severe TBI; DoC with high agitation; monitoring nociceptive burden, response to analgesia and longitudinal trajectories of nociception independent of consciousness fluctuations

Abbreviations: BINAM, Brain Injury Nociception Assessment Measure; BPS, Behavioural Pain Scale; CPOT, Critical‐Care Pain Observation Tool; CRS‐R, Coma Recovery Scale‐Revised; NCS, Nociception Coma Scale; NCS‐R, Nociception Coma Scale‐Revised; PAINAD, Pain Assessment in Advanced Dementia Scale.

Moving toward a more reflexive model, the Behavioural Pain Scale (BPS) conceptualizes nociception as a largely defensive biomechanical reaction (Payen et al. 2001). Facial grimacing, limb withdrawal and ventilatory patterns reflect segmental and bulbospinal defensive circuits. These behaviours map onto well‐characterized nociceptive reflex arcs, but the scale's applicability in patients with DoC is limited by sedation effects and the necessity of preserved motor pathways.

The Critical‐Care Pain Observation Tool (CPOT) develops this approach by integrating autonomic and ventilatory features with motor reactivity, reflecting the procedural nociception typical of ICU settings (Gélinas et al. 2006). The CPOT assumes that nociceptive stimulation may elicit reproducible motor and ventilatory responses even in sedated or non‐communicative patients. It integrates facial expression, body movement, muscle tension and ventilatory adaptation. However, its interpretation in DoC is limited by the potential dissociation between motor output and cortical processing.

A shift toward DoC‐specific frameworks begins with the NCS, which reframes nociception as ‘raw’ non‐cognitive reactivity (Schnakers et al. 2010). By separating facial, motor and vocal domains, NCS deliberately decouples nociceptive responses from higher‐order pain perception, offering a model in which spinal and brainstem mechanisms dominate. However, the inability to distinguish between reflexive and potentially cortically integrated responses motivated the revision of the scale. This revision culminates in the NCS‐R, which removes the poorly performing visual item and introduces standardized stimulus administration (Chatelle et al. 2012). The standard stimulus variant prioritizes inter‐rater reliability and cross‐session comparability, defining nociception as reactivity to a uniform nail‐bed pressure. In contrast, the personalized stimulus approach recognizes that clinically relevant nociception often emerges from individualized pain generators such as spasticity, heterotopic ossification or device‐related irritation. Multicenter evidence confirms that personalized stimulation increases sensitivity and ecological validity (Formisano et al. 2024), highlighting that nociceptive pathways can be differentially engaged depending on peripheral sensitization patterns.

Finally, the BINAM/BIDAL represents the most explicitly physiological model of nociception in severe brain injury (Whyte et al. 2020). Grounded in item‐response theory, BINAM captures a latent, unidimensional nociceptive drive that is demonstrably independent of the level of consciousness or agitation. By integrating autonomic outputs (blood pressure, heart rate, pupil diameter) with non‐volitional motor and facial behaviours, the scale operationalizes nociception as a bottom‐up, subcortically driven physiological process. Its sensitivity to analgesia and physical activity further validates its neurophysiological grounding, establishing BINAM as the first scale to quantify nociceptive load with psychometric interval properties.

Taken together, these instruments illustrate a conceptual progression: from affective distress (PAINAD) to defensive reflexive reactivity (BPS, CPOT) to DoC‐specific behavioural nociception (NCS/NCS‐R) and finally to latent physiological nociception (BINAM). Integrating them within a common framework clarifies both their complementarities and their mechanistic boundaries, supporting a more precise and theoretically coherent approach to pain and nociception assessment in non‐communicative patients.

### 4.1 Assessment of Pain: Technological Measures

Understanding how the pain matrix dynamically responds to nociceptive input increasingly relies on objective physiological measures capable of capturing its sensory, affective and autonomic dimensions. Beyond behavioural observation, a range of neurophysiological and autonomic biomarkers—such as EEG‐derived evoked potentials, fMRI‐based BOLD responses, PET metabolic activity, heart‐rate variability (HRV), galvanic skin response (GSR), pupillometry and composite multimodal indices—provide quantifiable signatures of activity within cortical and subcortical components of the pain matrix (Table 4). These measures allow researchers and clinicians to infer the integrity, modulation and hierarchical engagement of pain‐related networks even in individuals who cannot communicate, offering a more sensitive window into nociceptive processing and its potential integration into conscious experience. In healthy individuals, combined HRV and functional MRI studies during tonic cold pain or conditioned pain modulation paradigms show that low‐frequency HRV and indices of descending pain inhibition covary with functional connectivity between the PAG, ventromedial prefrontal cortex and anterior cingulate cortex (ACC), directly linking autonomic reactivity to central control of pain. Studies also indicate that reduced HRV, reflecting impaired parasympathetic and baroreflex function, is a robust feature of chronic pain conditions, supporting the view that pain and autonomic regulation share partially common neural mechanisms within the central autonomic network (CAN) (Cortese et al. 2020; Makovac et al. 2021; Riganello et al. 2023). In severe DoC, widespread disruption of thalamo‐cortical and fronto‐parietal networks would be expected to make direct access to the subjective dimension of pain impossible (Monti et al. 2010, 2015). Yet, converging behavioural, electrophysiological and neuroimaging evidence demonstrates residual nociceptive processing in a substantial subset of patients diagnosed with UWS or MCS. Laser‐evoked potential (LEP) studies show that some UWS and MCS patients exhibit preserved cortical LEP components and pain‐matrix activations comparable in latency and topography to healthy controls, suggesting that at least part of the cortical nociceptive system can remain functionally responsive despite absent or minimal overt behaviour (De Salvo et al. 2015; de Tommaso et al. 2013, 2015). Evidence from LEP and neuroimaging studies in DoC is heterogeneous. In some UWS/VS patients, only early components or restricted cortical responses have been observed, suggesting preserved afferent nociceptive processing but incomplete higher‐order integration. Laureys et al. reported restricted activation to noxious stimulation, mainly involving brainstem, thalamus and primary somatosensory cortex, in contrast to the broader network activation observed in healthy controls (Laureys et al. 2002). Similarly, de Tommaso et al. described preserved early laser‐evoked responses in some patients, but such responses do not necessarily imply conscious pain perception (de Tommaso et al. 2013, 2015). Broader and more integrated activations involving insula, ACC/MCC, frontoparietal or associative networks may increase the plausibility of conscious pain processing, particularly in MCS, but even in these cases interpretation remains inferential rather than definitive.

**TABLE 4:** Neurophysiological methods to assess pain/nociception in DoC.

Technique	Primary target	Main evidence in DoC	Clinical inference	Main limitation	Key references
LEPs and repeated‐stimulation LEP paradigms	Aδ‐mediated nociceptive cortical responses; early/late cortical components; temporal dynamics of habituation or sensitization	Some UWS/MCS patients show preserved LEP components or partial cortical responses to laser nociceptive stimulation; repeated paradigms may provide information on cortical excitability, habituation, salience updating or incomplete cortical integration	Evidence of residual nociceptive afferent/cortical processing; broader or late responses may increase the plausibility of higher‐order integration but do not demonstrate conscious pain	Protocols are heterogeneous; early components, late components and gamma/oscillatory responses should not be conflated; preserved LEPs are not sufficient evidence of conscious pain perception	de Tommaso et al. (2013), De Salvo et al. (2015), Naro et al. (2016)
EEG oscillatory responses/LEP‐induced gamma activity	Late cortical integration, oscillatory responses to nociceptive/salient stimulation	Gamma‐band or late EEG responses have been reported in selected DoC patients during nociceptive stimulation	Possible residual cortical integration or salience‐related processing	Low specificity; gamma activity may reflect arousal, attention, salience or nonspecific cortical activation rather than conscious pain	de Tommaso et al. (2015), Naro et al. (2016), Venturella et al. (2019)
fMRI/PET activation during noxious stimulation	Spatial distribution of pain‐related network activation; thalamo‐cortical and associative network engagement	UWS may show restricted activation involving brainstem, thalamus and S1; MCS may show broader activation including ACC/insula/frontoparietal regions	Degree of network integration; broader activation increases plausibility of pain‐related awareness but remains inferential	BOLD/PET activation is not equivalent to conscious experience; pain matrix regions are not pain‐specific	Laureys et al. (2002), Boly et al. (2008), Stender et al. (2014); Pistoia et al. (2016)
HRV/HRV entropy	CAN integrity; brain–heart coupling; autonomic complexity	HRV and entropy indices may discriminate UWS/MCS and correlate with CAN‐related connectivity or responses to noxious conditioning	Residual autonomic integration and nociceptive/autonomic reactivity	Not pain‐specific; affected by medication, lesion site, arousal, respiratory pattern, dysautonomia	Riganello et al. (2019), Cortese et al. (2020), Makovac et al. (2021)
Galvanic skin response/skin conductance	Sympathetic sudomotor reactivity; autonomic arousal	GSR changes during repeated noxious stimulation may indicate autonomic learning or salience‐related reactivity	Sympathetic response to nociceptive/salient stimulation	Low specificity; may reflect arousal, stress, thermoregulation or nonspecific sympathetic activation	Venturella et al. (2019), Cortese et al. (2020)
Pupillometry	LC–autonomic arousal; brainstem‐mediated nociceptive reactivity	Quantitative pupillometry may detect nociception‐related autonomic changes in unconscious/critically ill patients	Arousal/nociceptive load and brainstem autonomic responsiveness	Strongly affected by light, drugs, sedation, ocular factors and brainstem damage	Kyle and McNeil (2014), Fratino et al. (2021)
Multimodal behavioural–autonomic–neurophysiological protocols	Integrated inference across behavioural scales, EEG, autonomic signals, neuroimaging and clinical context	Combined approaches may improve stratification of residual nociceptive processing and guide individualized analgesic decisions	Probabilistic clinical inference about nociceptive burden and possible pain‐related awareness	Requires standardization, longitudinal validation and careful avoidance of overinterpretation	Schnakers and Zasler (2015), Cortese et al. (2020), Riganello et al. (2021)

Abbreviations: ACC, Anterior cingulate cortex; BOLD, Blood Oxygenation Level Dependent; CAN, central autonomic network; DoC, disorders of consciousness; EEG, Electroencephalography; fMRI, Functional magnetic resonance imaging; GSR, galvanic skin response; HRV, heart‐rate variability; LEP, Laser‐evoked potential; MCS, minimally conscious state; PET, Positron Emission Tomography; UWS: unresponsive wakefulness syndrome.

Clinically, the NCS quantifies motor, verbal, facial and visual responses to calibrated noxious stimuli, and their total scores correlate with cerebral glucose metabolism in fronto‐parietal regions and discriminate patients with a higher likelihood of pain perception, especially when a cut‐off score ≥ 5 is used (Chatelle, Thibaut, Bruno, et al. 2014). Recent multicentre work indicates that personalizing the painful stimulus, using clinically meaningful body sites instead of standard fingernail pressure, further increases NCS‐R sensitivity, underscoring the importance of individualized pain protocols in DoC (Formisano et al. 2024).

Autonomic indices such as HRV and HRV entropy provide a complementary window on pain processing, because they capture dynamic brain–heart interactions within the CAN during noxious versus innocuous stimulation and can discriminate between UWS, MCS and healthy controls (Cortese et al. 2020; Riganello et al. 2019). HRV‐entropy studies show lower baseline complexity in UWS compared with MCS, and higher entropy, together with galvanic skin response during trace conditioning to repeated noxious stimuli, identify a subgroup of behaviourally unresponsive patients who subsequently transition to MCS, consistent with implicit learning of nociceptive–affective contingencies (Cortese et al. 2020).

Overall, these data support a neuro‐ and psychophysiological framework in which, in DoC, pain‐related processing reflects residual integration across cortical nociceptive networks and the CAN, expressed through coupled behavioural, autonomic and neurophysiological signatures that can contribute to hypotheses regarding residual or covert consciousness, but cannot be considered direct evidence of conscious pain experience (Cortese et al. 2021).

## 5 Future Directions and Research Priorities

Future research on pain and nociception in disorders of consciousness should prioritize the development of clinically feasible multimodal assessment strategies that integrate behavioural scales with objective physiological and neurophysiological measures. However, beyond the technical integration of different modalities, a key conceptual challenge lies in aligning pain assessment with the patient's underlying capacity for conscious experience.

Recent advances in the study of DoC have demonstrated that behavioural responsiveness is an incomplete proxy for awareness, with a substantial proportion of patients showing evidence of covert consciousness despite the absence of overt motor output (Cascella et al. 2023; Velasco‐Muñoz et al. 2025). In parallel, research on thalamocortical dynamics, large‐scale brain network organization and neural complexity suggests that patients diagnosed as unresponsive wakefulness syndrome may retain varying degrees of residual integrative processing. These findings support a shift from categorical to graded and biologically informed models of consciousness. Within this framework, the interpretation of nociceptive responses requires caution. Neurophysiological, autonomic and neuroimaging signals may reflect sensory detection, salience processing or arousal‐related mechanisms, without necessarily implying the presence of a conscious and subjective experience of pain. Therefore, nociception and pain should not be considered equivalent, especially in the context of severely impaired consciousness.

Accordingly, future approaches should move toward an integrated multidimensional framework in which pain assessment is systematically embedded within the broader evaluation of consciousness capacity. Such a framework should combine: (i) standardized behavioural assessments, (ii) physiological and nociceptive markers and (iii) indices of large‐scale brain organization derived from electrophysiology, neuroimaging and complexity‐based metrics. From a clinical perspective, technologies such as EEG, autonomic monitoring, surface electromyography and wearable sensors may help detect changes in nociceptive reactivity over time and distinguish pain‐related responses from nonspecific fluctuations. Artificial intelligence may further support the integration of heterogeneous data sources, although its clinical implementation requires validation in large and representative cohorts (Rajpurkar et al. 2022; Bruschetta et al. 2022; Ahmed et al. 2025). More immediately applicable strategies include repeated structured assessments, individualized stimulation protocols and longitudinal monitoring of responses before and after analgesic interventions. Ultimately, integrating pain‐related measures with markers of consciousness capacity is essential not only to improve diagnostic accuracy, but also to guide ethically informed clinical decisions, including pain management, prognostic evaluation and end‐of‐life care in patients with severe brain injury (Formisano et al. 2024; Chatelle et al. 2012; Schnakers et al. 2010).

## 6 Conclusions

Pain and nociception should be considered clinically relevant dimensions in acquired brain injury, particularly in DoC, where behavioural, autonomic and neurophysiological responses may inform clinical management and rehabilitation planning. However, nociceptive processing, autonomic reactivity and conscious pain experience are distinct phenomena and should not be conflated. Manifestations such as autonomic dysregulation or features overlapping with paroxysmal sympathetic hyperactivity should also be interpreted as potential indicators of nociceptive burden or physiological distress, rather than as definitive evidence of conscious pain. Assessment should therefore rely on integrated, patient‐centered approaches combining behavioural observation, autonomic markers, clinical context and neurophysiological evidence when available. Given the possibility that some patients diagnosed as unresponsive may retain residual pain perception, careful evaluation remains both a clinical priority and an ethical responsibility.

This position paper calls for a cautious shift toward individualized, multimodal assessment strategies that may improve clinical interpretation, guide treatment and enhance care quality in patients with severe disorders of consciousness. In this framework, pain should be conceptualized as an emergent property of hierarchical brain organization, arising from the interaction between nociceptive processing and the residual capacity for large‐scale integrative activity supporting conscious experience.

## Author Contributions

Conceptualization, F.R., M.C., I.C. and A.C.; methodology, M.C., R.S.C. and A.C.; investigation, G.M., C.M., M.D.C.; resources, I.C.; data curation, F.S., M.E.P.; writing – original draft preparation, F.R., M.C., I.C. and A.C.; writing – review and editing, M.D.C, F.R., L.F.L.; supervision, R.S.C, D.B, I.C. and A.C.; All authors have read and agreed to the published version of the manuscript.

## Funding

The authors have nothing to report.

## Consent

This article does not contain any studies with human or animal subjects performed by any Human and Animal Rights and Informed Consent of the authors.

## Conflicts of Interest

The authors declare no conflicts of interest.
